# Predictive value of radiomics-based machine learning for the disease-free survival in breast cancer: a systematic review and meta-analysis

**DOI:** 10.3389/fonc.2023.1173090

**Published:** 2023-08-16

**Authors:** Dongmei Lu, Yuke Yan, Min Jiang, Shaoqin Sun, Haifeng Jiang, Yashan Lu, Wenwen Zhang, Xing Zhou

**Affiliations:** ^1^Department of Radiology, Gansu Provincial Hospital, Lanzhou, China; ^2^The Second Department of General Surgery, Gansu Provincial Hospital, Lanzhou, China

**Keywords:** breast cancer, radiomics, disease-free survival, prognosis, meta-analysis

## Abstract

**Purpose:**

This study summarized the previously-published studies regarding the use of radiomics-based predictive models for the identification of breast cancer-associated prognostic factors, which can help clinical decision-making and follow-up strategy.

**Materials and methods:**

This study has been pre-registered on PROSPERO. PubMed, Embase, Cochrane Library, and Web of Science were searched, from inception to April 23, 2022, for studies that used radiomics for prognostic prediction of breast cancer patients. Then the search was updated on July 18, 2023. Quality assessment was conducted using the Radiomics Quality Score, and meta-analysis was performed using R software.

**Results:**

A total of 975 articles were retrieved, and 13 studies were included, involving 5014 participants and 35 prognostic models. Among the models, 20 models were radiomics-based and the other 15 were based on clinical or pathological information. The primary outcome was Disease-free Survival (DFS). The retrieved studies were screened using LASSO, and Cox Regression was applied for modeling. The mean RQS was 18. The c-index of radiomics-based models for DFS prediction was 0.763 (95%CI 0.718-0.810) in the training set and 0.702 (95%CI 0.637-0.774) in the validation set. The c-index of combination models was 0.807 (95%CI0.736-0.885) in the training set and 0.840 (95%CI 0.794-0.888) in the validation set. There was no significant change in the c-index of DFS at 1, 2, 3, and over 5 years of follow-up.

**Conclusion:**

This study has proved that radiomics-based prognostic models are of great predictive performance for the prognosis of breast cancer patients. combination model shows significantly enhanced predictive performance.

**Systematic review registration:**

https://www.crd.york.ac.uk/PROSPERO/, identifier CRD42022332392.

## 1 Introduction

According to the statistical data released by the American Cancer Society (ACS) in 2022, breast cancer is the most prevalent malignancy and the fifth leading cause of cancer-related death among women (1), with a 5-year recurrence rate of 10.4% (2). The biological feature of breast cancer present high heterogeneity, which means that the treatment-response and prognosis of patients with the same type of breast cancer would be very different due to the molecular variances (3). It has been confirmed currently that axillary lymph node metastasis (ALNM), vascular invasion, hormone receptors expression, histological grades, and molecular subtypes are crucial factors for the recurrence risk and prognosis in breast cancer patients (4–6). However, these indicators are obtained only by biopsy and postoperative pathology, which is invasive. Therefore, studying the molecular heterogeneity of breast cancer is of significant clinical application value for risk stratification and long-term survival improvement in patients with breast cancer.

Radiomics refers to an emerging image quantitative analysis technique. In 2012, Lambin et al (7), defined it as a technology capable of obtaining high throughput feature from medical images. They also proposed that the application of radiomics should be combined with imaging, clinical, and pathological feature to obtain quantitative feature that could reflect changes in cancer in genetic and molecular levels, so as to speculate the protein genome and molecular phenotype and identify the intra-cancer and inter-cancer heterogeneity. Recent studies have shown that the heterogeneity of genome expression could be transformed into intra-cancer heterogeneity, which could be evaluated through imageology (8). Cancers with greater genome heterogeneity are more likely to induce drug resistance and early metastasis, and the prognosis of the patients would be poorer. This makes it feasible to apply radiomics for predicting the prognosis of cancer patients. The process of radiomics involves centralized separation of regions-of-interest (ROI) from imaging data sets and extraction of high-throughput image feature volume of interest (VOI) via automatic or semi-automatic software with specific imaging modes. These features can be roughly divided into morphological, first-level, second-level, and textural feature (9), and can be analyzed using multiple methods such as machine learning. Correlations of these feature with outcomes of clinical significance can be assessed to provide prediction endpoints of specific cancer. To date, the primary approaches to obtaining images in radiomics include Magnetic Resonance Imaging (MRI), Computed Tomography (CT), Ultrasound (US), Positron Emission Computed Tomography (PET-CT), and Mammography.

Radiomics, as a non-invasive technic, can reflect the overall feature of cancer, and can be performed repeatedly at different time points, which grants it unique advantages. Current radiomic studies regarding breast cancer mostly focus on benign and malignant differentiation (10), molecular typing and predicting (11, 12), preoperative ALNM evaluation (13), neoadjuvant chemotherapy assessment (14, 15), and recurrence prediction (16). Additionally, there are some studies combining radiomics with other disciplines (pathology, biomarkers, genomics, and proteomics) to explore the association between the feature of radiomics and the clinical outcomes such as disease-free survival (DFS) (17)and progression-free survival (PFS) (18). A machine learning model constructed based on clinical and pathological information can efficiently predict the DFS of early and advanced breast cancer. This has been basically proved by the test results of EndoPredict^®^ (EP) scores (c-index 0.7535), indicating that clinical features can convey part of the information expressed by genomic tests (19). In contrast, radiomics can provide abundant information on tumor phenotype and tumor microenvironment (20). Some studies have investigated whether machine learning models constructed based on clinical information and radiomics can accurately predict the prognosis and survival of breast cancer. However, the predictive accuracy of radiomics varies among these studies, and there remains a lack of evidence to summarize its predictive performance. The aim of this study is to assess the predictive value of radiomics-based models for the prognosis of patients with breast cancer.

## 2 Materials and methods

This study is conducted in strict accordance with the Preferred Reporting Items for Systematic Reviews and Meta-Analyses (PRISMA) 2020 statement (21), and has been registered on PROSPERO (URL: https://www.crd.york.ac.uk/PROSPERO/#recordDetails registration No. CRD42022332392). See **Supplementary Data Sheet 1** for details of PROSPERO registration.

### 2.1 Search strategy

PubMed, Embase, Cochrane Library, and Web of Science were searched, from database inception to April 23, 2022, for studies regarding radiomics (mammography, CT, US, or MRI) for predicting DFS in breast cancer patients. To ensure the integrity and comprehensiveness of data, the search was updated on July 18, 2023. The literature search was conducted by two reviewers independently. Search items were designed based on the combination of medical subject headings and free words, which mainly included “breast cancer”, “breast tumor”, “radiomics”, “prognosis”, and “DFS”. See **Table S1** for details of search strategy.

### 2.2 Inclusion and exclusion criteria

Inclusion criteria:

·Study subjects were female breast cancer patients
·Feature of radiomics were extracted from mammography, CT, US, or MRI, and a machine-learning risk model was constructed for prognostic prediction.
·Contained at least one of the following indicators to assess the predictive performance of the model: c-index, Receiver Operator Characteristic curve (ROC), Area Under the Curve (AUC), Sensitivity (SEN), Specificity (SPE), Accuracy, and Confusion Matrix.

Studies meeting the following criteria were excluded:

·Data unavailable.
·Un-published or repeatedly published studies.
·Other types of study: literature review, conference summary, case-report, comment, and animal study.

### 2.3 Study selection and data extraction

Endnote X9 was adopted for reference management. All retrieved articles were imported into Endnote X9. The duplicates were removed followed by titles and abstracts-reading to exclude irrelevant articles, and the full texts of the remaining articles were retrieved and read to identify studies to be included.

The following data were extracted: name of the first author, publication date, nationality, sample size, data sources, the major way for image-obtaining, software for extraction of regions of interest, selection of feature, and model construction method (see **Tables 1**–**3** for the detailed information). For each study, the overall c-index and c-index of different outcome-measurement time points in the training set and the validation set were extracted.

**Table 1:** Feature of participants.

No	Author	Year	Country	Dataset	Department	No	Age	Stages	treatment	Follow-up time
1	Yunfang Yu	2020	China	development	4 hospitals in China (randomly divided 7:3)	849	47^*^ (20, 22–35)	I-III	surgery and ALND	23.7(IQR:14.9-37.1)
				validation		365	47^*^ (20, 22–33, 36)			23.9(IQR:16.4-39.3)
2	Lang Xiong	2021	China	training	1 hospital in China (randomly divided)	372	49.10 ± 10.46	I-III	surgery, adjuvant therapy	48.99(IQR:44.42-62.98)
				validation		248	50.41 ± 10.76			
3	Ling Zhang	2020	China	training	1 hospital in China (randomly divided 2:1)	76	NA	NR	surgery, adjuvant therapy	44.4(range,5-93)
				validation		38	NA			
4	Bingqing Xia	2021	China	training	1 hospital in China another hospital in China	109	47.3 ± 11.1	II-III	surgery, NAC	54(range:1-101)
				validation		41	48.6 ± 13.3			48(range:1-88)
5	Feihong Yu	2021	China	training	the First Affiliated Hospital of Nanjing Medical University	216	NA	I-III	surgery, adjuvant therapy	53 (range,36–62)
				validation		108	NA			47(range,32–59)
				validation	remaining two institutions	162	NA			56 (range,37–65)
6	Sungwon Kim	2020	Korea	training	Severance Hospital, Yonsei University College of Medicine, Seoul, Korea	169	52.2 ± 12.5	NR	surgery, adjuvant therapy	48(range,5–80)
				validation		59	53.7 ± 11.5			
7	Hwan-ho Cho	2022	Switzerland	development	Samsung Medical Center	308	51.2 ± 10.5	NR	adjuvant therapy, breast-conserving surgery, mastectomy	84.1 (range,5–108)
				validation	Gil Hospital	147	49.1 ± 10.0			
8	Qin Li	2020	China	train	Fudan University Shanghai Cancer Center, Shanghai, China (randomly divided 7:3)	89	51.57 ± 10.88	NR	trastuzumab-based NAC conserving breast surgery or radical mastectomy	39.31(range,3–78)
				test		38	51.47 ± 9.40			
9	Xian Jiang	2020	China	training	West China Hospital, Sichuan University, Chengdu, China (randomly divided 2:1)	133	49.22 ± 9.87	I-III	surgery, adjuvant therapy	54.98 ± 21.72
				validation		67	47.79 ± 10.10			54.39 ± 22.63
10	Haoyu Wang	2022	China	Training	Shanghai Jiao Tong University School of Medicine, Shanghai, China	449	NA	I-III	surgical treatment, ALND	NR
				IV		113	NA			NR
				EV		40	NA		neoadjuvant therapy	NR
11	Hyunjin Park	2018	Korea	training	Samsung Medical Center, Sungkyunkwan University School of Medicine, Gangnam-gu, Korea.	194	50.46 ± 10.46	I-III	adjuvant therapy	54.2 (range,5–64)
				validation		100	52.34 ± 10.51			
12	Xuanyi Wang	2022	China	training	Fudan University Shanghai Cancer Center (randomly divided 1:1)	139	NA	II-III	adjuvant therapy	NR
				validation		139	NA			
				EV	The Cancer Imaging Archive (TCIA)	91	NA			
13	Jeongmin Lee	2022	Korea	Training Validation	Seoul Saint Mary’s Hospital(stratified random sampling)	111 44	34.94 ± 5.25 35.73 ± 3.48	I-IV	Surgery、radiation therapy、chemotherapy	55 (range,6–118)

D, development; EV, external validation; IV, internal validation; IQR, inter quartile range, NAC, neoadjuvant chemotherapy; NR, not report; ALND, axillary lymph node dissection, Follow-up time unit: months. *: median.

**Table 2:** Feature of radiomics.

No.	Author	Year	imaging	segmentation	VOI	ROI software	Feature extraction software	No. of features	Morphologic fearutes	First order features	Second order textural features
1	Yunfang Yu	2020	T1+C, T2WI, ADC	semi-automatically	Primary/ALN	3D Slicer4.10.2	PyRadiomics	2589	Yes	Yes	Yes
2	Lang Xiong	2021	US	manually	Primary	Photoshop	PyRadiomics	1209	Yes	Yes	Yes
3	Ling Zhang	2020	CT+C	semi-automatically	Primary	3D Slicer	pyradiomics	110	Yes (n=16)	Yes (n=19)	Yes (GLCM n=24, GLRLM n=16, GLSZM n=16, NGTDM n=5, GLDM n=14)
4	Bingqing Xia	2021	DCE-MRI	manually	Primary	3D Slicer	pyradiomics	1316	Yes (n=14)	Yes(n=930 + 72)	Yes (GLCM n=24, GLRLM n=16, NGTDM n=5, GLDM n=14) 75x4
5	Feihong Yu	2021	US	manually	Intratumoral + peritumoral	ITK–SNAP	pyradiomics	96	Yes (n=11)	Yes(n=17)	Yes (textural features n=68)
6	Sungwon Kim	2020	T2WI, T1WI+C	semi-automatically	Primary	MIPAV	PyRadiomics	2436	Yes	Yes	Yes
7	Hwan-ho Cho	2022	DCE	manually	Primary	NR	PyRadiomics				
8	Qin Li	2020	CE	manually	Primary	3D-Slicer	PyRadiomics	1037	Yes (n=14)	NA	Yes (textural features n=1023)
9	Xian Jiang	2020	Mammography	manually	Primary	NR	Local Image Feature Extraction	133	NA	NA	Yes (textural features n=133)(GLCM,NGLDM, GLRLM, GLZLM)
10	Haoyu Wang	2022	US	manually	Primary	ITK-SNAP	MATLAB	560	NA	Yes(n=16)	texture features (73 features) and wavelet features (356 features)
11	Hyunjin Park	2018	T2WI T1WI+C SubT1WI	manually	Primary	NR	MATLAB	156	Yes (n=8)	Yes(n=19)	Yes (texture features 18)
12	Xuanyi Wang	2022	ADC cT1WI, T2WI	manually	Primary	3D-Slicer	PyRadiomics	850	NR	NR	NR
13	Jeongmin Lee	2022	Sub DCE T1, ADC	semiautomatic	Primary	3D slicer	PyRadiomics	214	Yes	Yes	Yes GLCM(24 features), GLRLM (16 features), GLZLM (16 features), GLDM (14 features) and NGTDM (5 features)

US, ultrasound; DCE, dynamic contrast enhanced; C, contrast-enhanced; ADC, apparent diffusion coefficient; CT,c omputed tomography; VOI, volume of interest; GLRLM, Grey-Level Run Length Matrix; GLCM, Grey-Level Co-occurrence Matrix; GLSZM, Grey-Level Size Zone Matrix; NGTDM, Neighbourhood Grey Tone Difference Matrix; GLRLM, grey-level run length matrix; GLSZM, grey-level size zone matrix; GLDM: Gray-level Dependence Difference Matrix; NGLDM, neighboring gray level dependence matrix; GLZLM, gray-level zone length matrix; NA, not applicable; NR, not reported.

**Table 3:** Modeling and selection of radiomics feature.

No.	Author	Year	Dataset	No. of patients	Machine Learning Model	Model	Feature selection methods
1	Yunfang Yu	2020	development	849	LASSO-logistic regression model	radiomics	LASSO
			development	849	RF-Cox regression model	radiomics	RF
2	Lang Xiong	2021	training	372	Cox proportional hazards model	radiomics	Spearman correlation coefficients and Ward linkage, LASSO-COX
3	Ling Zhang	2020	training	76	Cox proportional hazard model	radiomics	LASSO Cox regression analysis
4	Bingqing Xia	2021	training	109	Cox proportional hazard model	radiomics	Forward stepwise regression
			training	109	Cox proportional hazard model	clinicoradiological	Forward stepwise regression
5	Feihong Yu	2021	training	216	LASSO-Cox	Radiomics	MRMR algorithm, LASSO-COX
6	Sungwon Kim	2020	training	169	Univariate and multivariate Cox proportional hazards models	Radiomic model	LASSO Cox regression analysis
7	Hwan-ho Cho	2022	training	169	Univariate and multivariate Cox proportional hazards models	combined clinicopathologic-radiomic (CCR) model	(LASSO) Cox regression analysis
			development	308	Cox-LASSO model	Radiomics DCE-MR	
			development	308	Cox-LASSO model	Radiomics Perfusion	
			development	308	L1-norm regularized Cox proportional hazard model	HRS only	Cox-LASSO
8	Qin Li	2020	training	89	univariate and multivariate Cox proportional hazards model	clinicoradiological based	Cox regression analysis
			training	89	univariate and multivariate Cox proportional hazards model	Radiomics- clinicoradiological based	Cox regression analysis
9	Xian Jiang	2020	training	133	Cox proportional hazards regression model	Radiomics	LASSO
10	Haoyu Wang	2022	Training	449	Logistic Regression	US only	Logistic Regression
						US + CP	
						US + SMOTEENN	
						CP + SMOTEENN	
						US + CP + SMOTEENN	
11	Hyunjin Park	2018	training	194	elastic net Cox regression	radiomic	elastic net
12	Xuanyi Wang	2022	training	139	Univariate and multivariate Cox	radiomics	LASSO
13	Jeongmin Lee	2022	training	111	LASSO-logistic regression	clinicoradiologic Radiomics	Univariate and multivariate Cox

LASSO, least absolute shrinkage and selection operator; RF, random forest; HRS=habitat risk score; US, ultrasound; CP, clinicopathological; SMOTEENN, (hybrid sampling method), combined over sampling technique SMOTE, (Synthetic Minority Oversampling Technique) and under sampling technique ENN, (Edited Nearest Neighbor). Radiomics-based model: the models constructed based on features extracted from US/CT/MRI/mammography; clinicopathological radiomics-based model: the models constructed based on the combination of radiomics features and required features such as age, menstrual status, TNM stage, histological grade, ER, PR, HER-2, The Ki67 index, axillary lymph node metastasis and the number of metastases, adjuvant treatment methods, and surgical methods.

Study screening and data extraction were conducted by two reviewers (LDM and YYK) independently, and the results were cross-checked by each other. Disagreements were settled by a third reviewer (ZX).

### 2.4 Quality assessment

Methodological quality and risk of bias of included studies were assessed by two reviewers (LDM and YYK) independently using the Radiomics Quality Score (RQS), and the results were cross-checked by each other. Disagreements were settled by a third reviewer (ZX).

### 2.5 Data synthesis and statistical analysis

Statistical analysis was performed using Stata 15.0 software. The accuracy of the models was assessed using c-statistic, and the 95% confidence interval (95%CI) was provided. Meta-analysis of c-statistic was performed using a random-effect model. Subgroup analysis based on different time points was conducted. A p value less than 0.05 indicated statistical significance.

## 3 Results

### 3.1 Study selection

**Figure 1:**
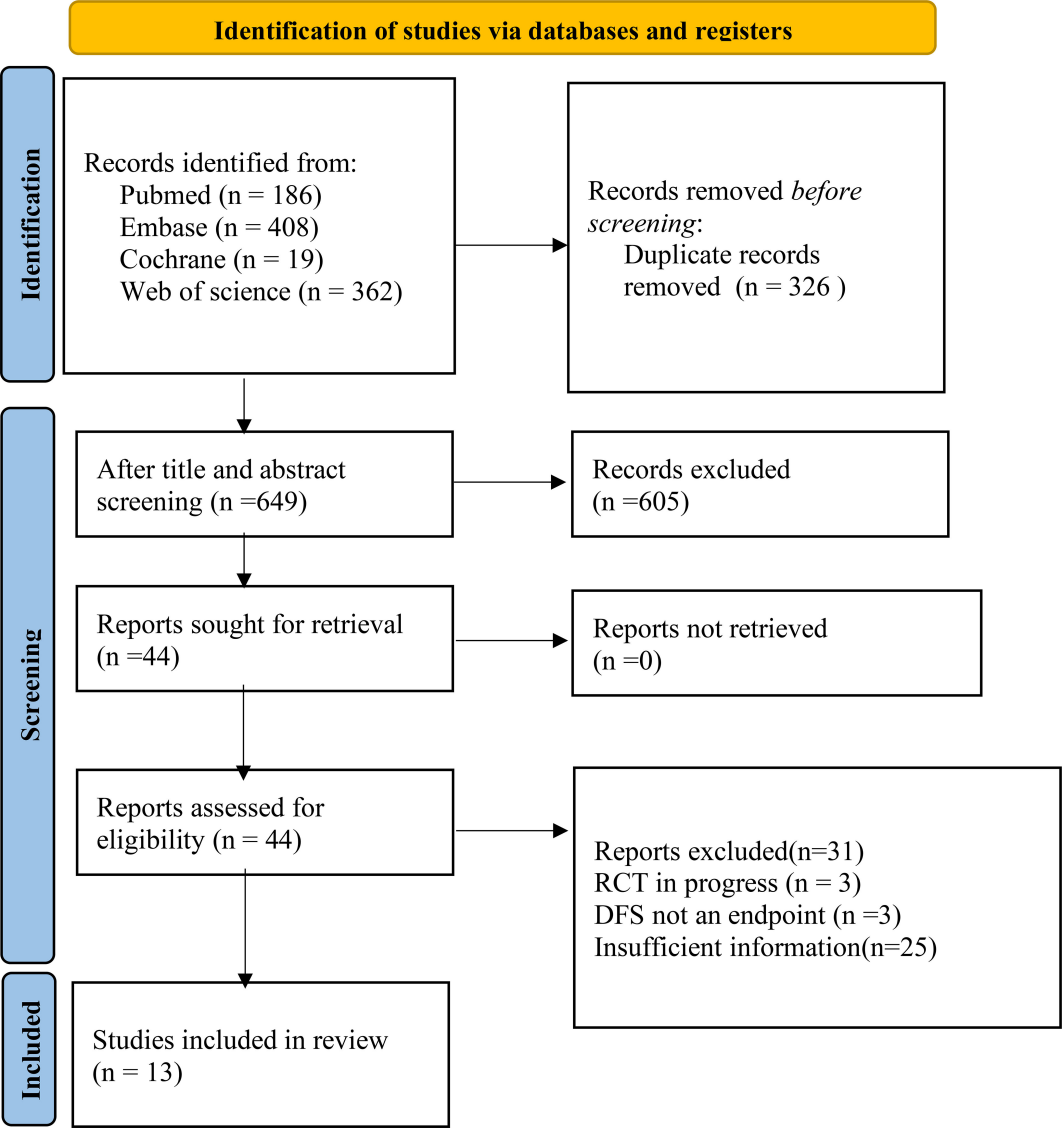
Structural regression for retrieved articles (from inception to July 18^th^, 2023) according to the PRISMA2020 guideline. Twelve studies regarding radiomics for after-treatment DFS prediction in breast cancer patients were finally included.

A total of 987articles were retrieved, and 13 studies were finally included, involving 35 datasets and 5014 participants (**Figure 1**, **Table 1**).

### 3.2 Feature of included studies

Among the included studies, the dataset of 1 study was from Switzerland (37), with datasets of 3 studies from Korea (38, 39), and 9 from China. The age of the participants ranged from 24 to 87 years, and the follow-up duration ranged from 1 to 101 months. Patients with triple-negative breast cancers (TNBC) accounted for 30.1% (1666), and those with Her-2 positive accounted for 2.53% (127). Participants in all the 13 included studies had received surgical treatment but maybe some differences are present in adjuvant treatments.

All 13 studies extracted radiomic feature from the baseline and pre-treatment images. Among the included studies, 1 study constructed the DFS-prediction model using Mammographic images and extracted tumor texture features in CC and MLO (40), 3 studies used US and the extracted feature included tumor size, morphology, peripheral and posterior acoustic feature, first-order statistical feature, 2D-based shape features, textural features, and wavelet features (41–43), and 8 studies adopted MRI and the involved sequences in extracted feature were: T1WI, T2WI, ADC, T1WI subtraction images and T1WI contrast-enhanced images. All these 7 studies used enhanced scanning sequences. As for the only 1 CT-based study, it also used contrast-enhanced scanning (44); There were 4 studies that performed predictive modeling for the DFS of TNBC patients, in which the study by Yu et al (42), extracted both the intra-tumoral and peritumoral radiomic feature.

Among the included studies, 11 studies performed overall extraction of cancer. The way of ROI extraction involved manual extraction and semi-automatic extraction. For software applied for ROI segmentation, 6 studies used 3Dslice and 2 used ITK-SNAP. For feature extraction of ROI, 10 studies applied PyRadiomics. The methods of characteristic-selection varied among the studies, including Intergroup Correlation Coefficient (ICC) and Lasso. The number of radiomic feature extracted from images ranged from 96 to 2589. Textural and morphological feature were the most common. Many models contained similar feature, including the Gray level co-occurrence matrix (GLCM) and Neighbouring Gray Tone Difference Matrix (NGTDM).

All 13 retrospective studies applied a machine learning model to predict the DFS of breast cancer patients. Cox regression was the most commonly used model. These studies had a different number of final characteristic parameters of model application, and different methods had been adopted for characteristic selection, including feature with significant ICC, Cox regression, or feature with significant p values in Kaplan-Meier analysis.

### 3.3 Quality assessment of included studies

The scoring items in RQS included: image capturing, radiomic feature extraction, data modeling, model validation, and data sharing. The total score ranged from -8 to 36. A score of -8 was defined as 0%, whereas 36 was 100% (45). The mean score of the 13 included studies was 18 (ranging from 15 to 26), defined as approximately 51.6% (**Table S2**).

### 3.4 Results of meta-analysis

**Figure 2:**
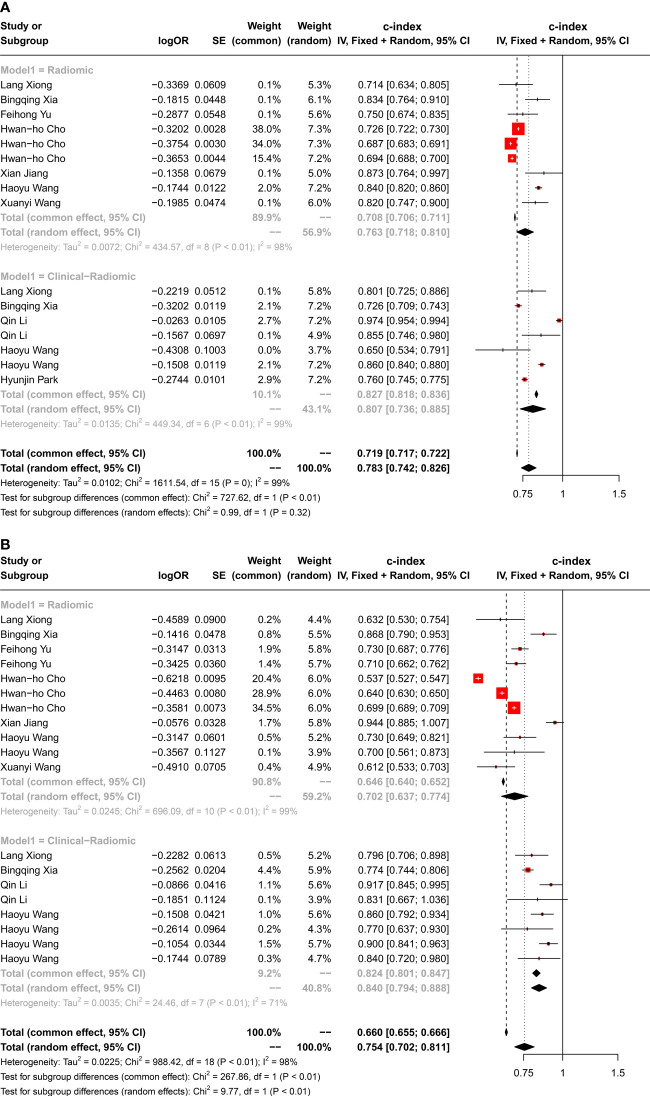
Forest plot of c-index in **(A)** training and **(B)** validation set prediction model.

For the 35 datasets included in this study, the c-index of radiomics-based models in DFS prediction was 0.763 (95%CI 0.718-0.810) in the training set and 0.702 (95%CI 0.637-0.774) in the validation set. The c-index of combination models was 0.807 (95%CI 0.736-0.885) in the training set and 0.840 (95%CI 0.794-0.888) in the validation set (**Figure 2**).

On the other hand, we summarized the c-indices of radiomics-based models at different time points, and the results showed that there was no difference between the c-index at different time points. It did not significantly decrease with time (**Table 4**).

**Table 4:** Summarized c-index of radiomic prediction models at different time points.

Follow time	training set		validation set	
	n	c-index (95%CI)	n	c-index (95%CI)
1 year	5	0.818 (0.772~0.866)	5	0.761 (0.625~0.926)
2 years	3	0.850 (0.802~0.900)	5	0.733 (0.633~0.848)
3 years	4	0.795 (0.725~0.873)	4	0.735 (0.636~0.848)
≥5years	8	0.770 (0.710~0.835)	11	0.745 (0.681~0.814)

## 4 Discussion

In this study, we have performed a systematic review and meta-analysis to assess the performance of radiomics-based prognostic models for predicting the survival of breast cancer patients receiving surgery but maybe some differences are present in adjuvant treatments. The results indicate that according to the summarized c-index, radiomics-based models would be of appropriate performance for predicting the DFS of breast cancer patients, and the performance could be enhanced in combination with patients` clinical and pathological backgrounds. Furthermore, no significant difference was observed in the predictive performance as the follow-up time extended.

DFS is the outcome measure of this meta-analysis, which is defined as the time from the completion of surgery and adjuvant therapy to the recurrence of the disease or death from the progression of the disease. Different treatment methods often cause different prognosis. For the early stage of breast cancer, there is no significant difference in the 5- and 10-year DFS between breast-conserving surgery and radical mastectomy (46). The local recurrence, distant metastasis, and overall survival are found to be improved in patients who received breast-conserving surgery followed by whole breast radiotherapy compared to those on radical mastectomy alone (47). Unhealthy lifestyles, such as drinking (48), postmenopausal obesity (49), family history are well-known risk factors for breast cancer recurrence. As the research deepens, demographical feature, clinical pathology, genetics, and serum tumor markers are applied for the prognostic assessment of breast cancer (36, 50–55). Therefore, in addition to the inclusion of clinical pathological information, the clinical pathological model constructed in this study also takes into account the treatment response of the patients. Univariate and multivariate analysis showed that tumor size, high pathological stage, lymphatic vessel invasion, high histological grading, non-pathological complete response (nPCR), young age of onset, and high Ki67 are all associated with poor DFS. Ki67 is an independent prognostic factor for breast cancer survival. For younger patients, it may be related to poor DFS, but for older patients (over 50 years old), it may have different impacts on their survival. High Ki-67 expression is associated with a higher risk of recurrence and poorer survival in patients with early breast cancer (22, 23).

Is it just the clinical pathological feature that determine the patient’s prognosis? Radiomics feature has been demonstrated to be an independent biomarker for predicting the prognosis of breast cancer [38]. As part of radiomics, texture analysis could quantify the spatial grey distribution features of the pixels and the spatial relationship between the pixels, so that it could reflect the intra-tumor heterogeneity (24). In This meta-analysis, the most applied radiomic model was texture analysis (10/13,83.3%), in which the most common texture was GLCM and NGTDM. GLCM is one of the most commonly used texture analysis methods that could describe information like change amplitude, adjacent interval, and direction, and it has been proven to evaluate tumor heterogeneity. GLCM encompasses 14 texture features, and the top five common features are energy, entropy, contrast, correlation, and inverse differential moment (IDM). Due to the differences in their mathematical definitions, these features reflect the texture heterogeneity of tumors from different aspects [27]. Gatenby et al. found that high entropy of T2WI (≥6.013, HR=9.84) and low entropy of T1WI-enhanced subtraction images (< 5.0.57, HR=4.55) were significantly associated with poor relapse-free survival [39]. NGTDM reflects the contrast, which is determined by the intensity of change between the target voxel and the surrounding adjacent voxels. Given the interaction between adjacent pixels, it is more suitable for quantifying tumor texture and heterogeneity [40, 41]. Tumors with poor prognosis tend to have higher contrast (25).

Rad score refers to a radiomic scoring system established through weighting the coefficients of each radiomic characteristic in the ROI and is a comprehensive indicator for the radiographic feature. In this meta-analysis, the c-index of the model with the peak performance has reached 0.974 (95%CI 0.954-0.994) (26). The results of this study showed that the clinical radiology model (MRI findings and clinical pathological variables) combined with the Rad score (c-index =0.974, 95% CI=0.954–0.994) outperformed simple clinical radiology model (c-index=0.855, 95% CI=0.739–0.971) in predicting disease-free survival. Xia et al. (27), constructed a training set Combined Radiomics Nomogram model based on the radiomic feature of MR contrast enhancement, Rad scores, and clinical pathological feature of 104 TNBC patients, with the c-index of 0.834 (95%CI 0.761-0.907), and the Clinicoradiological nomogram model based on radiomic feature of MR contrast enhancement yielded a c-index of 0.726 (0.709-0.734). These studies indicate that the RAD score can not only be used as an independent predictor for DFS and a biomarker for risk stratification in breast cancer, but is also helpful for developing more meticulous follow-up strategies for high-risk patients.

It is worth noting that T1W1-enhanced scanning sequences were involved in the 7 studies using MR for DFS prediction. The study by Hui et al. (25) found that with the decline in MRI-enhanced image texture parameters, the tumor heterogeneity was more significant, the risk of recurrence was higher, and the prognosis was worse. Lymphovascular invasion is associated with poorer prognosis in breast cancer (26). DCE-MRI-based radiomics feature is an independent risk factor for predicting lymphovascular invasion in patients with invasive ductal carcinoma (27). Hence, radiomics based on dynamic contrast-enhanced MR scan could provide more information and make the prognostic prediction of breast cancer more accurate, by reflecting the formation of tumor micro-vessels and the biological feature of the tumor (28). In 10 of the included studies (4, 20, 26, 27, 37–39, 41–43), the c-index of radiomics-based models constructed via MR ranged from 0.694 to 0.834, and this was overlapped with that of models constructed only using US (0.61 to 0.86). It remains to be elucidated whether MR-based radiomics would be more effective than US-based models.

Habitat analysis is the least explored field in the included studies, which aims to recognize different tumor sites or cell subsets. Conventional radiomics could measure to some extent the intra-tumoral heterogeneity. The measurement depends on a well-mixture of intra-tumoral heterogeneity but neglects the regional phenotypic variation (29). The sub-region segmentation technique focuses more on the intra-tumoral perfusion heterogeneity. Compared with the other 4 models, such as clinical models and radiomic models, a recurrence risk assessment model based on omics feature shows a better predictive performance (37). In addition, the spatial heterogeneity of each sub-region would be more important than the number of sub-regions. Perfusion heterogeneity defined by spatial heterogeneity among perfusion habitats is an independent predictor for DFS. Therefore, the quantification of perfusion heterogeneity is a potential method for prognostic prediction.

This study has some limitations to be improved and addressed in the future. All 13 included studies were retrospective studies with limited sample sizes, which could not meet the demands of radiomics in that the feature of high throughput require a large amount of data. This might induce selection bias. The lack of a “golden standard” for cancer segmentation and characteristic-extraction methods might also affect the reliability and repeatability. Only 3 of the 13 studies performed external validation for the model they constructed. External validation is more reliable than internal validation, and the data it produced is considered more independent.

On the other hand, the process of this meta-analysis also has limitations. One of the limitations of this study is the significant heterogeneity (*I²*=99%). The sources of heterogeneity might be associated with variances in imaging modality (such as CT, US, and MRI), manufacturer and model of the scanner, field intensity (1.5T, 3.0T), collection and inspection methods, and reconstruction parameters. Various parameters of scanners from distinct manufacturers and different image resolutions caused by different field strengths (1.5T, 3.0T) in scanners may affect the characteristic parameters of radiomics. Among the included studies, differences might also be attributed to the variances in operators` experiences and their understanding of the ROI scope. In addition, the heterogeneity might also be induced by differences in the parameters of the software used for extraction; However, this is an unavoidable limitation of the current systematic review regarding radiomics, and this study could not be spared. Another limitation is the different molecular subtypes of breast cancer, which might lead to a consequence that different radiomic feature are extracted. Some breast cancers with small volumes and multicentric/focal cancers have not been included (most of the studies extract feature from cancers with large volumes), which is difficult to be characterized by radiomic feature, leading to a deviation in the selection of radiomic feature. In addition, even though the incidence of breast cancer in the United States and India were reported to be relatively high, we did not find any eligible original studies published in these regions.

Currently, machine learning is increasingly applied in the medical field. Nonetheless, the interpretation of machine learning remains challenging. Some mathematical models, like support vector machines (SVM), random forests, probabilistic graphical models, reinforcement learning (RL), and deep learning (DL) neural networks, exhibit high diagnostic or predictive performance, but their interpretability is poor. However, the diagnostic or predictive performance of some interpretable machine learning methods is unsatisfying (30). Modeling variables are crucial for the performance of machine learning models. In recent years, modeling variables in clinical practice consist mainly of interpretable clinical features and some difficult-to-interpret image features (e.g., radiomics). Interpretability is a serious challenge in original research on radiomics, especially deep learning-based methods. Despite the high diagnostic or predictive performance, it is difficult to be widely used in clinical practice (31). In addition, it is difficult for radiomics to avoid the “curse of dimensionality”. Thus, interpretability should also be considered in the selection of dimensionality reduction methods. In our study, the variables of the included studies are screened using the rank sum test of texture features and LASSO regression, and the models are mainly based on Cox regression, which shows relatively good interpretability. Some studies have shown that other interpretable machine learning methods appear to be more accurate than traditional Cox regression in predicting the prognosis of breast cancer (32, 33). However, Cox regression, as one of the few modeling methods for survival analysis, is one of the first choices for modeling when the time variable needs to be considered. Meanwhile, follow-up studies on the prognosis of breast cancer are desired to explore early alternative outcome events and use interpretable machine learning methods of non-survival analysis to improve the predictive performance for the prognosis of breast cancer.

Given the limitations mentioned above, future studies should focus on prospective radiomic study design, as well as the standardization of imaging, stability of high-throughput feature, characteristic-selection method, and classifier. At the same time, the feature and models of the collected external prospective datasets should be validated to better explain the spatial variation and heterogeneity of voxel intensity in tumors for imaging training set and validation set, which is particularly important in multicentric studies (34, 35). In recent years, studies on multimodality and multi-omics have also achieved preliminary progress. Different imaging approaches contain different cancer information. The combination of image feature with different modalities could improve the predictive performance of the model. In addition to imaging information, cancer pathology, metabolic pathway, and gene expression also provide cancer information, which is crucial in revealing cancer heterogeneity. Future studies can be conducted based on the combination of radiomics, pathomics, proteomics, and genomics to develop radiomic feature with a biological basis. Besides, the study of breast cancer imaging is a deep intersection of medicine and computer artificial intelligence. Deep learning learns the feature of cancers from the data itself, which avoids the errors caused by the subjectivity of manual operation, making it more effective and reliable.

## 5 Conclusion

Radiomics is an interdisciplinary field that integrates multiple disciplines such as imageology, oncology, and machine learning. Radiomic feature (such as intensity, morphology, texture, or wavelet) provide information on cancer phenotype and microenvironment, which is complementary to other relevant data sources (including clinical, treatment-related, or genetic data) (29). The results of this study indicate that prognostic model performance could be enhanced after combining patients` clinical and pathological results. Therefore, we can try to construct a prediction model based on radiomics to effectively evaluate the prognosis of breast cancer patients in combination with the practical experience of clinicians. However, studies in this field have indeed a long way to go due to the heterogeneity and imaging complexity of breast cancer. More prospective and multi-centric studies with large cohorts are needed.

## Author contributions

DL: Conceptualization, Methodology, Formal analysis, Investigation, Data Curation, Writing - Original Draft, Writing - Review & Editing, Project administration. YY: Conceptualization, Methodology, Formal analysis, Resources, Data Curation, Writing - Original Draft, Writing - Review & Editing, Project administration. MJ: Methodology, Software, Formal analysis, Data Curation, Writing - Original Draft, Writing - Review & Editing, Supervision. SS: Software, Validation, Writing - Original Draft, Writing - Review & Editing, Visualization, Supervision. HJ: Software, Validation, Writing - Original Draft, Writing - Review & Editing, Visualization. YL: Validation, Writing - Original Draft, Writing - Review & Editing, Visualization. WZ: Validation, Writing - Original Draft, Writing - Review & Editing, Funding acquisition. XZ: Conceptualization, Methodology, Investigation, Resources, Data Curation, Writing - Original Draft, Writing - Review & Editing, Project administration. All authors contributed to the article and approved the submitted version

## Supplementary material

Supplementary DATA SHEET 1 PROSPERO registration details.
