# Supplementary material for: Predictive value of radiomics-based machine learning for the disease-free survival in breast cancer: a systematic review and meta-analysis
Source: Front Oncol. 2023 Aug 16;13:1173090. doi: 10.3389/fonc.2023.1173090 (PMC10469000; doi:10.3389/fonc.2023.1173090)
Supplement: Supplementary DATA SHEET 1 — PROSPERO registration details. [file DataSheet_1.pdf]

National Institute for Health Research

UNIVERSITY of York
Centre for Reviews and Dissemination

Systematic review

A list of fields that can be edited in an update can be found here

1. * Review title.

Give the title of the review in English

Predictive value of a radiomics-based machine learning model for breast cancer prognosis: a systematic review and meta-analysis

2. Original language title.

For reviews in languages other than English, give the title in the original language. This will be displayed with the English language title.

3. * Anticipated or actual start date.

Give the date the systematic review started or is expected to start.

25/04/2022

4. * Anticipated completion date.

Give the date by which the review is expected to be completed.

25/06/2022

5. * Stage of review at time of this submission.

This field uses answers to initial screening questions. It cannot be edited until after registration.

Tick the boxes to show which review tasks have been started and which have been completed.

Update this field each time any amendments are made to a published record.

The review has not yet started: No

Review stage	Started	Completed
Preliminary searches	Yes	Yes
Piloting of the study selection process	Yes	Yes
Formal screening of search results against eligibility criteria	Yes	No
Data extraction	No	No
Risk of bias (quality) assessment	No	No
Data analysis	No	No

Provide any other relevant information about the stage of the review here.

6. * Named contact.

The named contact is the guarantor for the accuracy of the information in the register record. This may be any member of the review team.

Dongmei LU

Email salutation (e.g. "Dr Smith" or "Joanne") for correspondence:

Mrs LU

7. * Named contact email.

Give the electronic email address of the named contact.

346083151@qq.com

8. Named contact address

Give the full institutional/organisational postal address for the named contact.

No. 204, Donggang West Road, Chengguan District, Lanzhou City, Gansu Province, China

9. Named contact phone number.

Give the telephone number for the named contact, including international dialling code.

18909461556

10. * Organisational affiliation of the review.

Full title of the organisational affiliations for this review and website address if available. This field may be

completed as 'None' if the review is not affiliated to any organisation.

Gansu Provincial People's Hospital

Organisation web address:

https://www.gsyy.cn/

11. * Review team members and their organisational affiliations.

Give the personal details and the organisational affiliations of each member of the review team. Affiliation refers to groups or organisations to which review team members belong. **NOTE: email and country now MUST be entered for each person, unless you are amending a published record.**

Mrs Dongmei LU. Gansu Provincial People's Hospital
Mrs Lirong ZHANG. The 940th Hospital of the Joint Logistics Support Force of the Chinese People's Liberation Army
Mrs Wenwen ZHANG. Gansu Provincial People's Hospital

12. * Funding sources/sponsors.

Details of the individuals, organizations, groups, companies or other legal entities who have funded or sponsored the review.

individuals

Grant number(s)

State the funder, grant or award number and the date of award

13. * Conflicts of interest.

List actual or perceived conflicts of interest (financial or academic).

None

14. Collaborators.

Give the name and affiliation of any individuals or organisations who are working on the review but who are not listed as review team members. **NOTE: email and country must be completed for each person, unless you are amending a published record.**

15. * Review question.

State the review question(s) clearly and precisely. It may be appropriate to break very broad questions down into a series of related more specific questions. Questions may be framed or refined using PI(E)COS or similar where relevant.

Through data mining and machine learning, radiomics can obtain high-dimensional features that cannot be recognized by the naked eye in medical images and apply them to clinical decision support systems. Currently, it is widely used in the field of tumors. In the breast, it is mainly used in the identification of benign

and malignant breast cancer, molecular typing and evaluation of the efficacy of neoadjuvant chemotherapy. Studies have shown that radiomics has broad research prospects in the prognosis and prediction of breast cancer.

16. * Searches.

State the sources that will be searched (e.g. Medline). Give the search dates, and any restrictions (e.g. language or publication date). Do NOT enter the full search strategy (it may be provided as a link or attachment below.)

Literature retrieval was performed on embase, PubMed, web of knowledge and the Cochrane Library. The retrieval strategy used subject headings + free words. There was no language restriction for literature retrieval. The retrieval time was from the establishment of the database to April 25, 2022.

17. URL to search strategy.

Upload a file with your search strategy, or an example of a search strategy for a specific database, (including the keywords) in pdf or word format. In doing so you are consenting to the file being made publicly accessible. Or provide a URL or link to the strategy. Do NOT provide links to your search **results**.

Alternatively, upload your search strategy to CRD in pdf format. Please note that by doing so you are consenting to the file being made publicly accessible.

Yes I give permission for this file to be made publicly available

18. * Condition or domain being studied.

Give a short description of the disease, condition or healthcare domain being studied in your systematic review.

Radiomics has a wide range of applications in the medical field, especially in the field of tumors; in breast cancer, the application of omics analysis to non-invasive prediction and evaluation of prognosis has great prospects, but there is no unified conclusion yet, so this Meta analyze.

19. * Participants/population.

Specify the participants or populations being studied in the review. The preferred format includes details of both inclusion and exclusion criteria.

This systematic review included patients with breast cancer, and the inclusion criteria were as follows: 1) Breast cancer diagnosed by pathological examination; 2) Complete imaging examination two weeks before surgery; 3) No history of other systemic malignant tumors; 4) No history of malignancy before imaging examination or chemoradiotherapy;

The exclusion criteria were as follows: 1) no complete imaging data; 2) poor image quality; 3) incomplete follow-up data; 4) local recurrence and distant metastases.

20. * Intervention(s), exposure(s).

Give full and clear descriptions or definitions of the interventions or the exposures to be reviewed. The preferred format includes details of both inclusion and exclusion criteria.

This systematic review includes breast cancer prognostic indicators: 1) disease-free survival: the interval time between the surgery and recurrence or breast cancer-related death; 2) Progress-free survival, PFS; 3) overall survival, OS

21. * Comparator(s)/control.

Where relevant, give details of the alternatives against which the intervention/exposure will be compared (e.g. another intervention or a non-exposed control group). The preferred format includes details of both inclusion and exclusion criteria.

DFS was considered as the end point of the present study, which was defined as the interval time between the surgery and recurrence or breast cancer-related death, which ever came first. Recurrence means locoregional recurrence, distant metastasis, or contralateral breast cancer. Physical examination, histopathology, and imaging modalities such as US, computed tomography, MRI were used to demonstrated the recurrence. At the last follow-up, patients without an event and/or died of non breast cancer related events were censored.

22. * Types of study to be included.

Give details of the study designs (e.g. RCT) that are eligible for inclusion in the review. The preferred format includes both inclusion and exclusion criteria. If there are no restrictions on the types of study, this should be stated.

Studies included in this systematic review include: randomized controlled trials, cohort studies, case-control studies, nested case-control studies

23. Context.

Give summary details of the setting or other relevant characteristics, which help define the inclusion or exclusion criteria.

24. * Main outcome(s).

Give the pre-specified main (most important) outcomes of the review, including details of how the outcome is defined and measured and when these measurement are made, if these are part of the review inclusion criteria.

sensitivity , specificity, C-index

Measures of effect

Please specify the effect measure(s) for you main outcome(s) e.g. relative risks, odds ratios, risk difference, and/or 'number needed to treat.

The outcome indicators of this systematic review include: sensitivity (95% confidence interval), specificity (95% confidence interval), C-index (area under the curve at each time point of follow-up time)

25. * Additional outcome(s).

List the pre-specified additional outcomes of the review, with a similar level of detail to that required for main outcomes. Where there are no additional outcomes please state 'None' or 'Not applicable' as appropriate to the review

The secondary outcome indicators of this systematic review include: Progress free survival, PFS; 3) overall survival, OS; omics model types include: LASSO–Cox regression model, The random forest–Cox regression model, Stepwise regression;Variable screening methods include: 1) least absolute shrinkage and selection operator (LASSO); 2) Intraclass correlation coefficients (ICC)

Measures of effect

Please specify the effect measure(s) for you additional outcome(s) e.g. relative risks, odds ratios, risk difference, and/or 'number needed to treat.

26. * Data extraction (selection and coding).

Describe how studies will be selected for inclusion. State what data will be extracted or obtained. State how this will be done and recorded.

1) Search the literature according to the subject heading + free word search strategy, then import the literature into the endnote, and incorporate it into the original research by reading the research purpose, abstract and full text,

2) Two trained diagnostic physicians (lirong, Zhang, 5 years of diagnosis experience, Wenwen, Zhang, 8 years of diagnosis experience) extracted relevant data and established relevant data tables.

27. * Risk of bias (quality) assessment.

State which characteristics of the studies will be assessed and/or any formal risk of bias/quality assessment tools that will be used.

Two researchers (L.Z, W.Z) evaluate the methodological quality of the included research with the radio quality score (RQs), and conduct interactive inspection after completion. If there is any dispute, the third researcher shall be invited to assist in the adjudication. Among them, RQs is a unique quality evaluation tool of radiomics. The original research design was scored from 16 items, with a full score of 36.

28. * Strategy for data synthesis.

Describe the methods you plan to use to synthesise data. This **must not be generic text** but should be **specific to your review** and describe how the proposed approach will be applied to your data. If meta-analysis is planned, describe the models to be used, methods to explore statistical heterogeneity, and software package to be used.

The statistical analysis of this study is in stata15 Completed in 0. We first performed grouping analysis according to the follow-up time, and summarized the ROC and its 95% confidence interval of the prediction model. Then, the bivariate mixed effect model was used to evaluate the prediction accuracy of machine learning method for DFS and OS at different time points of the system. The sensitivity and specificity of the model summary, P 0.05, were considered to be statistically different.

29. * Analysis of subgroups or subsets.

State any planned investigation of 'subgroups'. Be clear and specific about which type of study or participant will be included in each group or covariate investigated. State the planned analytic approach.

Subgroup analysis was performed according to the evaluation time points (1 year, 3 years, 5 years) of the included literature outcome indicators

30. * Type and method of review.

Select the type of review, review method and health area from the lists below.

Type of review

Cost effectiveness

No

Diagnostic

Yes

Epidemiologic

No

Individual patient data (IPD) meta-analysis

No

Intervention

No

Living systematic review

No

Meta-analysis

Yes

Methodology

No

Narrative synthesis

No

Network meta-analysis

No

Pre-clinical

No

Prevention

No

Prognostic

No

Prospective meta-analysis (PMA)

No

Review of reviews

No

Service delivery

No

Synthesis of qualitative studies

No

Systematic review

Yes

Other

No

Health area of the review

Alcohol/substance misuse/abuse

No

Blood and immune system

No

Cancer

Yes

Cardiovascular

No

Care of the elderly

No

Child health

No

Complementary therapies

No

COVID-19

No

Crime and justice

No

Dental

No

Digestive system

No

Ear, nose and throat

No

Education

No

Endocrine and metabolic disorders

No

Eye disorders

No

General interest

No

Genetics

No

Health inequalities/health equity

No

Infections and infestations

No

International development

No

Mental health and behavioural conditions

No

Musculoskeletal

No

Neurological

No

Nursing

No

Obstetrics and gynaecology

No

Oral health

No

Palliative care

No

Perioperative care

No

Physiotherapy

No

Pregnancy and childbirth

No

Public health (including social determinants of health)

No

Rehabilitation

No

Respiratory disorders

No

Service delivery

No

Skin disorders

No

Social care

No

Surgery

No

Tropical Medicine

No

Urological

No

Wounds, injuries and accidents

No

Violence and abuse

No

31. Language.

Select each language individually to add it to the list below, use the bin icon to remove any added in error.

English

There is not an English language summary

32. * Country.

Select the country in which the review is being carried out. For multi-national collaborations select all the countries involved.

China

33. Other registration details.

Name any other organisation where the systematic review title or protocol is registered (e.g. Campbell, or The Joanna Briggs Institute) together with any unique identification number assigned by them. If extracted

data will be stored and made available through a repository such as the Systematic Review Data Repository (SRDR), details and a link should be included here. If none, leave blank.

34. Reference and/or URL for published protocol.

If the protocol for this review is published provide details (authors, title and journal details, preferably in Vancouver format)

Add web link to the published protocol.

Or, upload your published protocol here in pdf format. Note that the upload will be publicly accessible.

No I do not make this file publicly available until the review is complete

Please note that the information required in the PROSPERO registration form must be completed in full even if access to a protocol is given.

35. Dissemination plans.

Do you intend to publish the review on completion?

No

Give brief details of plans for communicating review findings.?

36. Keywords.

Give words or phrases that best describe the review. Separate keywords with a semicolon or new line. Keywords help PROSPERO users find your review (keywords do not appear in the public record but are included in searches). Be as specific and precise as possible. Avoid acronyms and abbreviations unless these are in wide use.

Keywords for this systematic review include: omics, breast cancer, prognosis

37. Details of any existing review of the same topic by the same authors.

If you are registering an update of an existing review give details of the earlier versions and include a full bibliographic reference, if available.

38. * Current review status.

Update review status when the review is completed and when it is published.New registrations must be ongoing so this field is not editable for initial submission.

Please provide anticipated publication date

Review_Ongoing

39. Any additional information.

Provide any other information relevant to the registration of this review.

40. Details of final report/publication(s) or preprints if available.

Leave empty until publication details are available OR you have a link to a preprint (NOTE: this field is not editable for initial submission). List authors, title and journal details preferably in Vancouver format.

Give the link to the published review or preprint.
