# Supplementary material for: Predictive value of radiomics-based machine learning for the disease-free survival in breast cancer: a systematic review and meta-analysis
Source: Front Oncol. 2023 Aug 16;13:1173090. doi: 10.3389/fonc.2023.1173090 (PMC10469000; doi:10.3389/fonc.2023.1173090)
Supplement: Supplementary file 2 [file Table_1.docx]

**Table S1.** Search Strategy

**1. PubMed**

| Search number | Query | | Search Details | Results |
| --- | --- | --- | --- | --- |
| 8 | ((("Breast Neoplasms"[Mesh]) OR (((((((((((((((((((((((((((((((((((((Breast Neoplasms[Title/Abstract]) OR (Breast Neoplasms[Title/Abstract])) OR (Breast Neoplasms[Title/Abstract])) OR (Breast Neoplasms[Title/Abstract])) OR (Tumor, Breast[Title/Abstract])) OR (Tumor, Breast[Title/Abstract])) OR (Tumor, Breast[Title/Abstract])) OR (Tumor, Breast[Title/Abstract])) OR (Cancer, Breast[Title/Abstract])) OR (Mammary Cancer[Title/Abstract])) OR (Cancer, Mammary[Title/Abstract])) OR (Cancers, Mammary[Title/Abstract])) OR (Mammary Cancers[Title/Abstract])) OR (Malignant Neoplasm of Breast[Title/Abstract])) OR (Breast Malignant Neoplasm[Title/Abstract])) OR (Breast Malignant Neoplasms[Title/Abstract])) OR (Malignant Tumor of Breast[Title/Abstract])) OR (Breast Malignant Tumor[Title/Abstract])) OR (Breast Malignant Tumors[Title/Abstract])) OR (Cancer of Breast[Title/Abstract])) OR (Cancer of the Breast[Title/Abstract])) OR (Mammary Carcinoma, Human[Title/Abstract])) OR (Carcinoma, Human Mammary[Title/Abstract])) OR (Carcinomas, Human Mammary[Title/Abstract])) OR (Human Mammary Carcinomas[Title/Abstract])) OR (Mammary Carcinomas, Human[Title/Abstract])) OR (Human Mammary Carcinoma[Title/Abstract])) OR (Mammary Neoplasms, Human[Title/Abstract])) OR (Human Mammary Neoplasm[Title/Abstract])) OR (Human Mammary Neoplasms[Title/Abstract])) OR (Neoplasm, Human Mammary[Title/Abstract])) OR (Neoplasms, Human Mammary[Title/Abstract])) OR (Mammary Neoplasm, Human[Title/Abstract])) OR (Breast Carcinoma[Title/Abstract])) OR (Breast Carcinomas[Title/Abstract])) OR (Carcinoma, Breast[Title/Abstract])) OR (Carcinomas, Breast[Title/Abstract]))) AND (((((Radiomics[Title/Abstract]) OR (radiomic[Title/Abstract])) OR (radiogenomic[Title/Abstract])) OR (radiomics-based[Title/Abstract])) OR (radiomic signature[Title/Abstract]))) AND ((Prognosis[MeSH Terms]) OR ((((((((((((((((((((((((Prognoses[Title/Abstract]) OR (Prognoses[Title/Abstract])) OR (Prognostic Factors[Title/Abstract])) OR (Prognostic Factor[Title/Abstract])) OR (Factor, Prognostic[Title/Abstract])) OR (Factors, Prognostic[Title/Abstract])) OR (Overall survival[Title/Abstract])) OR (OS[Title/Abstract])) OR (metastasis[Title/Abstract])) OR (Disease-Free Survival[Title/Abstract])) OR (Disease Free Survival[Title/Abstract])) OR (Survival, Disease-Free[Title/Abstract])) OR (Survival, Disease Free[Title/Abstract])) OR (DFS[Title/Abstract])) OR (Progression-Free Survival[Title/Abstract])) OR (Progression Free Survival[Title/Abstract])) OR (Survival, Progression-Free[Title/Abstract])) OR (Event-Free Survival[Title/Abstract])) OR (Event Free Survival[Title/Abstract])) OR (Survival, Event-Free[Title/Abstract])) OR (PFS[Title/Abstract])) OR (Mortality[Title/Abstract])) OR (Death[Title/Abstract])) OR (Mortalities[Title/Abstract]))) | | ("Breast Neoplasms"[MeSH Terms] OR ("Breast Neoplasms"[Title/Abstract] OR "Breast Neoplasms"[Title/Abstract] OR "Breast Neoplasms"[Title/Abstract] OR "Breast Neoplasms"[Title/Abstract] OR "tumor breast"[Title/Abstract] OR "tumor breast"[Title/Abstract] OR "tumor breast"[Title/Abstract] OR "tumor breast"[Title/Abstract] OR "cancer breast"[Title/Abstract] OR "mammary cancer"[Title/Abstract] OR "cancer mammary"[Title/Abstract] OR "cancers mammary"[Title/Abstract] OR "mammary cancers"[Title/Abstract] OR "malignant neoplasm of breast"[Title/Abstract] OR "breast malignant neoplasm"[Title/Abstract] OR "breast malignant neoplasms"[Title/Abstract] OR "malignant tumor of breast"[Title/Abstract] OR "breast malignant tumor"[Title/Abstract] OR "breast malignant tumors"[Title/Abstract] OR "cancer of breast"[Title/Abstract] OR "cancer of the breast"[Title/Abstract] OR "mammary carcinoma human"[Title/Abstract] OR (("Carcinoma"[MeSH Terms] OR "Carcinoma"[All Fields] OR "Carcinomas"[All Fields] OR "carcinoma s"[All Fields]) AND "human mammary"[Title/Abstract]) OR (("Carcinoma"[MeSH Terms] OR "Carcinoma"[All Fields] OR "Carcinomas"[All Fields] OR "carcinoma s"[All Fields]) AND "human mammary"[Title/Abstract]) OR "human mammary carcinomas"[Title/Abstract] OR (("mammaries"[All Fields] OR "mammary glands, human"[MeSH Terms] OR ("Mammary"[All Fields] AND "glands"[All Fields] AND "Human"[All Fields]) OR "human mammary glands"[All Fields] OR "Mammary"[All Fields] OR "Breast"[MeSH Terms] OR "Breast"[All Fields]) AND "carcinomas human"[Title/Abstract]) OR "human mammary carcinoma"[Title/Abstract] OR (("mammaries"[All Fields] OR "mammary glands, human"[MeSH Terms] OR ("Mammary"[All Fields] AND "glands"[All Fields] AND "Human"[All Fields]) OR "human mammary glands"[All Fields] OR "Mammary"[All Fields] OR "Breast"[MeSH Terms] OR "Breast"[All Fields]) AND "neoplasms human"[Title/Abstract]) OR (("human s"[All Fields] OR "humans"[MeSH Terms] OR "humans"[All Fields] OR "Human"[All Fields]) AND "mammary neoplasm"[Title/Abstract]) OR "human mammary neoplasms"[Title/Abstract] OR (("neoplasm s"[All Fields] OR "Neoplasms"[MeSH Terms] OR "Neoplasms"[All Fields] OR "Neoplasm"[All Fields]) AND "human mammary"[Title/Abstract]) OR (("neoplasm s"[All Fields] OR "Neoplasms"[MeSH Terms] OR "Neoplasms"[All Fields] OR "Neoplasm"[All Fields]) AND "human mammary"[Title/Abstract]) OR (("mammaries"[All Fields] OR "mammary glands, human"[MeSH Terms] OR ("Mammary"[All Fields] AND "glands"[All Fields] AND "Human"[All Fields]) OR "human mammary glands"[All Fields] OR "Mammary"[All Fields] OR "Breast"[MeSH Terms] OR "Breast"[All Fields]) AND "neoplasm human"[Title/Abstract]) OR "breast carcinoma"[Title/Abstract] OR "breast carcinomas"[Title/Abstract] OR "carcinoma breast"[Title/Abstract] OR "carcinomas breast"[Title/Abstract])) AND ("Radiomics"[Title/Abstract] OR "radiomic"[Title/Abstract] OR "radiogenomic"[Title/Abstract] OR "radiomics-based"[Title/Abstract] OR "radiomic signature"[Title/Abstract]) AND ("prognosis"[MeSH Terms] OR ("Prognoses"[Title/Abstract] OR "Prognoses"[Title/Abstract] OR "prognostic factors"[Title/Abstract] OR "prognostic factor"[Title/Abstract] OR "factor prognostic"[Title/Abstract] OR "factors prognostic"[Title/Abstract] OR "overall survival"[Title/Abstract] OR "OS"[Title/Abstract] OR "metastasis"[Title/Abstract] OR "disease free survival"[Title/Abstract] OR "disease free survival"[Title/Abstract] OR "survival disease free"[Title/Abstract] OR "survival disease free"[Title/Abstract] OR "DFS"[Title/Abstract] OR "progression free survival"[Title/Abstract] OR "progression free survival"[Title/Abstract] OR "survival progression free"[Title/Abstract] OR "event free survival"[Title/Abstract] OR "event free survival"[Title/Abstract] OR "survival event free"[Title/Abstract] OR "PFS"[Title/Abstract] OR "Mortality"[Title/Abstract] OR "Death"[Title/Abstract] OR "Mortalities"[Title/Abstract])) | 130 |
| 7 | (Prognosis[MeSH Terms]) OR ((((((((((((((((((((((((Prognoses[Title/Abstract]) OR (Prognoses[Title/Abstract])) OR (Prognostic Factors[Title/Abstract])) OR (Prognostic Factor[Title/Abstract])) OR (Factor, Prognostic[Title/Abstract])) OR (Factors, Prognostic[Title/Abstract])) OR (Overall survival[Title/Abstract])) OR (OS[Title/Abstract])) OR (metastasis[Title/Abstract])) OR (Disease-Free Survival[Title/Abstract])) OR (Disease Free Survival[Title/Abstract])) OR (Survival, Disease-Free[Title/Abstract])) OR (Survival, Disease Free[Title/Abstract])) OR (DFS[Title/Abstract])) OR (Progression-Free Survival[Title/Abstract])) OR (Progression Free Survival[Title/Abstract])) OR (Survival, Progression-Free[Title/Abstract])) OR (Event-Free Survival[Title/Abstract])) OR (Event Free Survival[Title/Abstract])) OR (Survival, Event-Free[Title/Abstract])) OR (PFS[Title/Abstract])) OR (Mortality[Title/Abstract])) OR (Death[Title/Abstract])) OR (Mortalities[Title/Abstract])) | | "prognosis"[MeSH Terms] OR "Prognoses"[Title/Abstract] OR "Prognoses"[Title/Abstract] OR "prognostic factors"[Title/Abstract] OR "prognostic factor"[Title/Abstract] OR "factor prognostic"[Title/Abstract] OR "factors prognostic"[Title/Abstract] OR "overall survival"[Title/Abstract] OR "OS"[Title/Abstract] OR "metastasis"[Title/Abstract] OR "disease free survival"[Title/Abstract] OR "disease free survival"[Title/Abstract] OR "survival disease free"[Title/Abstract] OR "survival disease free"[Title/Abstract] OR "DFS"[Title/Abstract] OR "progression free survival"[Title/Abstract] OR "progression free survival"[Title/Abstract] OR "survival progression free"[Title/Abstract] OR "event free survival"[Title/Abstract] OR "event free survival"[Title/Abstract] OR "survival event free"[Title/Abstract] OR "PFS"[Title/Abstract] OR "Mortality"[Title/Abstract] OR "Death"[Title/Abstract] OR "Mortalities"[Title/Abstract] | 3,428,616 |
| 6 | (((((((((((((((((((((((Prognoses[Title/Abstract]) OR (Prognoses[Title/Abstract])) OR (Prognostic Factors[Title/Abstract])) OR (Prognostic Factor[Title/Abstract])) OR (Factor, Prognostic[Title/Abstract])) OR (Factors, Prognostic[Title/Abstract])) OR (Overall survival[Title/Abstract])) OR (OS[Title/Abstract])) OR (metastasis[Title/Abstract])) OR (Disease-Free Survival[Title/Abstract])) OR (Disease Free Survival[Title/Abstract])) OR (Survival, Disease-Free[Title/Abstract])) OR (Survival, Disease Free[Title/Abstract])) OR (DFS[Title/Abstract])) OR (Progression-Free Survival[Title/Abstract])) OR (Progression Free Survival[Title/Abstract])) OR (Survival, Progression-Free[Title/Abstract])) OR (Event-Free Survival[Title/Abstract])) OR (Event Free Survival[Title/Abstract])) OR (Survival, Event-Free[Title/Abstract])) OR (PFS[Title/Abstract])) OR (Mortality[Title/Abstract])) OR (Death[Title/Abstract])) OR (Mortalities[Title/Abstract]) | | "Prognoses"[Title/Abstract] OR "Prognoses"[Title/Abstract] OR "prognostic factors"[Title/Abstract] OR "prognostic factor"[Title/Abstract] OR "factor prognostic"[Title/Abstract] OR "factors prognostic"[Title/Abstract] OR "overall survival"[Title/Abstract] OR "OS"[Title/Abstract] OR "metastasis"[Title/Abstract] OR "disease free survival"[Title/Abstract] OR "disease free survival"[Title/Abstract] OR "survival disease free"[Title/Abstract] OR "survival disease free"[Title/Abstract] OR "DFS"[Title/Abstract] OR "progression free survival"[Title/Abstract] OR "progression free survival"[Title/Abstract] OR "survival progression free"[Title/Abstract] OR "event free survival"[Title/Abstract] OR "event free survival"[Title/Abstract] OR "survival event free"[Title/Abstract] OR "PFS"[Title/Abstract] OR "Mortality"[Title/Abstract] OR "Death"[Title/Abstract] OR "Mortalities"[Title/Abstract] | 2,072,645 |
| 5 | Prognosis[MeSH Terms] | | "prognosis"[MeSH Terms] | 1,838,037 |
| 4 | ((((Radiomics[Title/Abstract]) OR (radiomic[Title/Abstract])) OR (radiogenomic[Title/Abstract])) OR (radiomics-based[Title/Abstract])) OR (radiomic signature[Title/Abstract]) | | "Radiomics"[Title/Abstract] OR "radiomic"[Title/Abstract] OR "radiogenomic"[Title/Abstract] OR "radiomics-based"[Title/Abstract] OR "radiomic signature"[Title/Abstract] | 5,771 |
| 3 | ("Breast Neoplasms"[Mesh]) OR (((((((((((((((((((((((((((((((((((((Breast Neoplasms[Title/Abstract]) OR (Breast Neoplasms[Title/Abstract])) OR (Breast Neoplasms[Title/Abstract])) OR (Breast Neoplasms[Title/Abstract])) OR (Tumor, Breast[Title/Abstract])) OR (Tumor, Breast[Title/Abstract])) OR (Tumor, Breast[Title/Abstract])) OR (Tumor, Breast[Title/Abstract])) OR (Cancer, Breast[Title/Abstract])) OR (Mammary Cancer[Title/Abstract])) OR (Cancer, Mammary[Title/Abstract])) OR (Cancers, Mammary[Title/Abstract])) OR (Mammary Cancers[Title/Abstract])) OR (Malignant Neoplasm of Breast[Title/Abstract])) OR (Breast Malignant Neoplasm[Title/Abstract])) OR (Breast Malignant Neoplasms[Title/Abstract])) OR (Malignant Tumor of Breast[Title/Abstract])) OR (Breast Malignant Tumor[Title/Abstract])) OR (Breast Malignant Tumors[Title/Abstract])) OR (Cancer of Breast[Title/Abstract])) OR (Cancer of the Breast[Title/Abstract])) OR (Mammary Carcinoma, Human[Title/Abstract])) OR (Carcinoma, Human Mammary[Title/Abstract])) OR (Carcinomas, Human Mammary[Title/Abstract])) OR (Human Mammary Carcinomas[Title/Abstract])) OR (Mammary Carcinomas, Human[Title/Abstract])) OR (Human Mammary Carcinoma[Title/Abstract])) OR (Mammary Neoplasms, Human[Title/Abstract])) OR (Human Mammary Neoplasm[Title/Abstract])) OR (Human Mammary Neoplasms[Title/Abstract])) OR (Neoplasm, Human Mammary[Title/Abstract])) OR (Neoplasms, Human Mammary[Title/Abstract])) OR (Mammary Neoplasm, Human[Title/Abstract])) OR (Breast Carcinoma[Title/Abstract])) OR (Breast Carcinomas[Title/Abstract])) OR (Carcinoma, Breast[Title/Abstract])) OR (Carcinomas, Breast[Title/Abstract])) | | "Breast Neoplasms"[MeSH Terms] OR ("Breast Neoplasms"[Title/Abstract] OR "Breast Neoplasms"[Title/Abstract] OR "Breast Neoplasms"[Title/Abstract] OR "Breast Neoplasms"[Title/Abstract] OR "tumor breast"[Title/Abstract] OR "tumor breast"[Title/Abstract] OR "tumor breast"[Title/Abstract] OR "tumor breast"[Title/Abstract] OR "cancer breast"[Title/Abstract] OR "mammary cancer"[Title/Abstract] OR "cancer mammary"[Title/Abstract] OR "cancers mammary"[Title/Abstract] OR "mammary cancers"[Title/Abstract] OR "malignant neoplasm of breast"[Title/Abstract] OR "breast malignant neoplasm"[Title/Abstract] OR "breast malignant neoplasms"[Title/Abstract] OR "malignant tumor of breast"[Title/Abstract] OR "breast malignant tumor"[Title/Abstract] OR "breast malignant tumors"[Title/Abstract] OR "cancer of breast"[Title/Abstract] OR "cancer of the breast"[Title/Abstract] OR "mammary carcinoma human"[Title/Abstract] OR (("Carcinoma"[MeSH Terms] OR "Carcinoma"[All Fields] OR "Carcinomas"[All Fields] OR "carcinoma s"[All Fields]) AND "human mammary"[Title/Abstract]) OR (("Carcinoma"[MeSH Terms] OR "Carcinoma"[All Fields] OR "Carcinomas"[All Fields] OR "carcinoma s"[All Fields]) AND "human mammary"[Title/Abstract]) OR "human mammary carcinomas"[Title/Abstract] OR (("mammaries"[All Fields] OR "mammary glands, human"[MeSH Terms] OR ("Mammary"[All Fields] AND "glands"[All Fields] AND "Human"[All Fields]) OR "human mammary glands"[All Fields] OR "Mammary"[All Fields] OR "Breast"[MeSH Terms] OR "Breast"[All Fields]) AND "carcinomas human"[Title/Abstract]) OR "human mammary carcinoma"[Title/Abstract] OR (("mammaries"[All Fields] OR "mammary glands, human"[MeSH Terms] OR ("Mammary"[All Fields] AND "glands"[All Fields] AND "Human"[All Fields]) OR "human mammary glands"[All Fields] OR "Mammary"[All Fields] OR "Breast"[MeSH Terms] OR "Breast"[All Fields]) AND "neoplasms human"[Title/Abstract]) OR (("human s"[All Fields] OR "humans"[MeSH Terms] OR "humans"[All Fields] OR "Human"[All Fields]) AND "mammary neoplasm"[Title/Abstract]) OR "human mammary neoplasms"[Title/Abstract] OR (("neoplasm s"[All Fields] OR "Neoplasms"[MeSH Terms] OR "Neoplasms"[All Fields] OR "Neoplasm"[All Fields]) AND "human mammary"[Title/Abstract]) OR (("neoplasm s"[All Fields] OR "Neoplasms"[MeSH Terms] OR "Neoplasms"[All Fields] OR "Neoplasm"[All Fields]) AND "human mammary"[Title/Abstract]) OR (("mammaries"[All Fields] OR "mammary glands, human"[MeSH Terms] OR ("Mammary"[All Fields] AND "glands"[All Fields] AND "Human"[All Fields]) OR "human mammary glands"[All Fields] OR "Mammary"[All Fields] OR "Breast"[MeSH Terms] OR "Breast"[All Fields]) AND "neoplasm human"[Title/Abstract]) OR "breast carcinoma"[Title/Abstract] OR "breast carcinomas"[Title/Abstract] OR "carcinoma breast"[Title/Abstract] OR "carcinomas breast"[Title/Abstract]) | 338,672 |
| 2 | ((((((((((((((((((((((((((((((((((((Breast Neoplasms[Title/Abstract]) OR (Breast Neoplasms[Title/Abstract])) OR (Breast Neoplasms[Title/Abstract])) OR (Breast Neoplasms[Title/Abstract])) OR (Tumor, Breast[Title/Abstract])) OR (Tumor, Breast[Title/Abstract])) OR (Tumor, Breast[Title/Abstract])) OR (Tumor, Breast[Title/Abstract])) OR (Cancer, Breast[Title/Abstract])) OR (Mammary Cancer[Title/Abstract])) OR (Cancer, Mammary[Title/Abstract])) OR (Cancers, Mammary[Title/Abstract])) OR (Mammary Cancers[Title/Abstract])) OR (Malignant Neoplasm of Breast[Title/Abstract])) OR (Breast Malignant Neoplasm[Title/Abstract])) OR (Breast Malignant Neoplasms[Title/Abstract])) OR (Malignant Tumor of Breast[Title/Abstract])) OR (Breast Malignant Tumor[Title/Abstract])) OR (Breast Malignant Tumors[Title/Abstract])) OR (Cancer of Breast[Title/Abstract])) OR (Cancer of the Breast[Title/Abstract])) OR (Mammary Carcinoma, Human[Title/Abstract])) OR (Carcinoma, Human Mammary[Title/Abstract])) OR (Carcinomas, Human Mammary[Title/Abstract])) OR (Human Mammary Carcinomas[Title/Abstract])) OR (Mammary Carcinomas, Human[Title/Abstract])) OR (Human Mammary Carcinoma[Title/Abstract])) OR (Mammary Neoplasms, Human[Title/Abstract])) OR (Human Mammary Neoplasm[Title/Abstract])) OR (Human Mammary Neoplasms[Title/Abstract])) OR (Neoplasm, Human Mammary[Title/Abstract])) OR (Neoplasms, Human Mammary[Title/Abstract])) OR (Mammary Neoplasm, Human[Title/Abstract])) OR (Breast Carcinoma[Title/Abstract])) OR (Breast Carcinomas[Title/Abstract])) OR (Carcinoma, Breast[Title/Abstract])) OR (Carcinomas, Breast[Title/Abstract]) | | "breast neoplasms"[Title/Abstract] OR "breast neoplasms"[Title/Abstract] OR "breast neoplasms"[Title/Abstract] OR "breast neoplasms"[Title/Abstract] OR "tumor breast"[Title/Abstract] OR "tumor breast"[Title/Abstract] OR "tumor breast"[Title/Abstract] OR "tumor breast"[Title/Abstract] OR "cancer breast"[Title/Abstract] OR "mammary cancer"[Title/Abstract] OR "cancer mammary"[Title/Abstract] OR "cancers mammary"[Title/Abstract] OR "mammary cancers"[Title/Abstract] OR "malignant neoplasm of breast"[Title/Abstract] OR "breast malignant neoplasm"[Title/Abstract] OR "breast malignant neoplasms"[Title/Abstract] OR "malignant tumor of breast"[Title/Abstract] OR "breast malignant tumor"[Title/Abstract] OR "breast malignant tumors"[Title/Abstract] OR "cancer of breast"[Title/Abstract] OR "cancer of the breast"[Title/Abstract] OR "mammary carcinoma human"[Title/Abstract] OR (("Carcinoma"[MeSH Terms] OR "Carcinoma"[All Fields] OR "Carcinomas"[All Fields] OR "carcinoma s"[All Fields]) AND "human mammary"[Title/Abstract]) OR (("Carcinoma"[MeSH Terms] OR "Carcinoma"[All Fields] OR "Carcinomas"[All Fields] OR "carcinoma s"[All Fields]) AND "human mammary"[Title/Abstract]) OR "human mammary carcinomas"[Title/Abstract] OR (("mammaries"[All Fields] OR "mammary glands, human"[MeSH Terms] OR ("Mammary"[All Fields] AND "glands"[All Fields] AND "Human"[All Fields]) OR "human mammary glands"[All Fields] OR "Mammary"[All Fields] OR "Breast"[MeSH Terms] OR "Breast"[All Fields]) AND "carcinomas human"[Title/Abstract]) OR "human mammary carcinoma"[Title/Abstract] OR (("mammaries"[All Fields] OR "mammary glands, human"[MeSH Terms] OR ("Mammary"[All Fields] AND "glands"[All Fields] AND "Human"[All Fields]) OR "human mammary glands"[All Fields] OR "Mammary"[All Fields] OR "Breast"[MeSH Terms] OR "Breast"[All Fields]) AND "neoplasms human"[Title/Abstract]) OR (("human s"[All Fields] OR "humans"[MeSH Terms] OR "humans"[All Fields] OR "Human"[All Fields]) AND "mammary neoplasm"[Title/Abstract]) OR "human mammary neoplasms"[Title/Abstract] OR (("neoplasm s"[All Fields] OR "Neoplasms"[MeSH Terms] OR "Neoplasms"[All Fields] OR "Neoplasm"[All Fields]) AND "human mammary"[Title/Abstract]) OR (("neoplasm s"[All Fields] OR "Neoplasms"[MeSH Terms] OR "Neoplasms"[All Fields] OR "Neoplasm"[All Fields]) AND "human mammary"[Title/Abstract]) OR (("mammaries"[All Fields] OR "mammary glands, human"[MeSH Terms] OR ("Mammary"[All Fields] AND "glands"[All Fields] AND "Human"[All Fields]) OR "human mammary glands"[All Fields] OR "Mammary"[All Fields] OR "Breast"[MeSH Terms] OR "Breast"[All Fields]) AND "neoplasm human"[Title/Abstract]) OR "breast carcinoma"[Title/Abstract] OR "breast carcinomas"[Title/Abstract] OR "carcinoma breast"[Title/Abstract] OR "carcinomas breast"[Title/Abstract] | 53,159 |
| 1 | "Breast Neoplasms"[Mesh] | Most Recent | "Breast Neoplasms"[MeSH Terms] | 325,228 |

**2. Embase**

| No. | Query | Result |
| --- | --- | --- |
| #1 | ('breast'/exp OR breast) AND ('tumor'/exp OR tumor) | 712154 |
| #2 | 'bilateral breast neoplasm':ab,ti OR 'bilateral breast tumor':ab,ti OR 'bilateral breast tumour':ab,ti OR 'breast gland tumor':ab,ti OR 'breast gland tumour':ab,ti OR 'breast mass; breast neoplasia':ab,ti OR 'breast neoplasm':ab,ti OR 'breast neoplasms':ab,ti OR 'breast neoplasms, male':ab,ti OR 'breast tumorigenesis':ab,ti OR 'breast tumour':ab,ti OR 'female breast neoplasm':ab,ti OR 'female breast tumor':ab,ti OR 'female breast tumour':ab,ti OR 'male breast neoplasm':ab,ti OR 'male breast tumor':ab,ti OR 'male breast tumour':ab,ti OR 'mamma tumor':ab,ti OR 'mamma tumour':ab,ti OR 'mammary gland neoplasia':ab,ti OR 'mammary gland neoplasm':ab,ti OR 'mammary gland tumor':ab,ti OR 'mammary gland tumorigenesis':ab,ti OR 'mammary gland tumour':ab,ti OR 'mammary neoplasia':ab,ti OR 'mammary neoplasm':ab,ti OR 'mammary neoplasms':ab,ti OR 'mammary tumor':ab,ti OR 'mammary tumor cell':ab,ti OR 'mammary tumorigenesis':ab,ti OR 'mammary tumour':ab,ti OR 'mammary tumour cell':ab,ti OR 'mass in the breast':ab,ti OR 'masses in the breast':ab,ti OR 'neoplasia of the breast':ab,ti OR 'neoplasm of the breast':ab,ti OR 'neoplasm of the mammary gland':ab,ti OR 'neoplastic breast':ab,ti OR 'neoplastic mammary':ab,ti OR 'neoplastic mammary gland':ab,ti OR 'tumor of the breast':ab,ti OR 'tumor of the female breast':ab,ti OR 'tumor of the male breast':ab,ti OR 'tumor of the mammary gland':ab,ti OR 'tumorigenesis of the breast':ab,ti OR 'tumorigenesis of the mammary gland':ab,ti OR 'tumour of the male breast':ab,ti OR 'unilateral breast neoplasm':ab,ti OR 'unilateral breast neoplasms':ab,ti OR 'unilateral breast tumor':ab,ti | 20438 |
| #3 | #1 OR #2 | 715563 |
| #4 | 'radiomics':ab,ti OR 'radiomic':ab,ti OR 'radiogenomic':ab,ti OR 'radiomics-based':ab,ti OR 'radiomic signature':ab,ti | 7985 |
| #5 | 'prognosis'/exp OR prognosis | 1117162 |
| #6 | 'prognoses':ab,ti OR 'prognostic factors':ab,ti OR 'prognostic factor':ab,ti OR 'factor, prognostic':ab,ti OR 'factors, prognostic':ab,ti OR 'overall survival':ab,ti OR 'os':ab,ti OR 'metastasis':ab,ti OR 'disease-free survival':ab,ti OR 'disease free survival':ab,ti OR 'survival, disease-free':ab,ti OR 'survival, disease free':ab,ti OR 'dfs':ab,ti OR 'progression-free survival':ab,ti OR 'progression free survival':ab,ti OR 'survival, progression-free':ab,ti OR 'event-free survival':ab,ti OR 'event free survival':ab,ti OR 'survival, event-free':ab,ti OR 'pfs':ab,ti OR 'mortality':ab,ti OR 'death':ab,ti OR 'mortalities':ab,ti | 3001142 |
| #7 | #5 OR #6 | 3651580 |
| #8 | #3 AND #4 AND #7 | 246 |

**3. Cochrane**

| No. | Query |
| --- | --- |
| #1 | MeSH descriptor: [Breast Neoplasms] explode all trees |
| #2 | (Breast Neoplasm):ti,ab,kw OR (Neoplasm, Breast):ti,ab,kw OR (Breast Tumors):ti,ab,kw OR (Breast Tumor):ti,ab,kw OR (Tumor, Breast):ti,ab,kw OR (Tumors, Breast):ti,ab,kw OR (Neoplasms, Breast):ti,ab,kw OR (Breast Cancer):ti,ab,kw OR (Cancer, Breast):ti,ab,kw OR (Mammary Cancer):ti,ab,kw OR (Cancer, Mammary):ti,ab,kw OR (Cancers, Mammary):ti,ab,kw OR (Mammary Cancers):ti,ab,kw OR (Malignant Neoplasm of Breast):ti,ab,kw OR (Breast Malignant Neoplasm):ti,ab,kw OR (Breast Malignant Neoplasms):ti,ab,kw OR (Malignant Tumor of Breast):ti,ab,kw OR (Breast Malignant Tumor):ti,ab,kw OR (Breast Malignant Tumors):ti,ab,kw OR (Cancer of Breast):ti,ab,kw OR (Cancer of the Breast):ti,ab,kw OR (Mammary Carcinoma, Human):ti,ab,kw OR (Carcinoma, Human Mammary):ti,ab,kw OR (Carcinomas, Human Mammary):ti,ab,kw OR (Human Mammary Carcinomas):ti,ab,kw OR (Mammary Carcinomas, Human):ti,ab,kw OR (Human Mammary Carcinoma):ti,ab,kw OR (Mammary Neoplasms, Human):ti,ab,kw OR (Human Mammary Neoplasm):ti,ab,kw OR (Human Mammary Neoplasms):ti,ab,kw OR (Neoplasm, Human Mammary):ti,ab,kw OR (Neoplasms, Human Mammary):ti,ab,kw OR (Mammary Neoplasm, Human):ti,ab,kw OR (Breast Carcinoma):ti,ab,kw OR (Breast Carcinomas):ti,ab,kw OR (Carcinoma, Breast):ti,ab,kw OR (Carcinomas, Breast):ti,ab,kw |
| #3 | #1 or #2 |
| #4 | (Radiomics ):ti,ab,kw OR (radiomic):ti,ab,kw OR (radiogenomic):ti,ab,kw OR (radiomics-based):ti,ab,kw OR (radiomic signature):ti,ab,kw |
| #5 | MeSH descriptor: [Prognosis] explode all trees |
| #6 | (Prognoses):ti,ab,kw OR (Prognostic Factors):ti,ab,kw OR (Prognostic Factor):ti,ab,kw OR (Factor, Prognostic):ti,ab,kw OR (Factors, Prognostic):ti,ab,kw OR (Overall survival):ti,ab,kw OR (OS):ti,ab,kw OR (metastasis):ti,ab,kw OR (Disease-Free Survival):ti,ab,kw OR (Disease Free Survival):ti,ab,kw OR (Survival, Disease-Free):ti,ab,kw OR (Survival, Disease Free):ti,ab,kw OR (DFS):ti,ab,kw OR (Progression-Free Survival):ti,ab,kw OR (Progression Free Survival):ti,ab,kw OR (Survival, Progression-Free):ti,ab,kw OR (Event-Free Survival):ti,ab,kw OR (Event Free Survival):ti,ab,kw OR (Survival, Event-Free):ti,ab,kw OR (PFS):ti,ab,kw OR (Mortality):ti,ab,kw OR (Death):ti,ab,kw OR (Mortalities):ti,ab,kw |
| #7 | #5 or #6 |
| #8 | #3 and #4 and #7 |

**4. Web of Science**

| No. | Query |
| --- | --- |
| #4 | #3 AND #2 AND #1 |
| #3 | TS=(Breast Neoplasms OR Breast Neoplasm OR Neoplasm, Breast OR Breast Tumors OR Breast Tumor OR Tumor, Breast OR Tumors, Breast OR Neoplasms, Breast OR Breast Cancer OR Cancer, Breast OR Mammary Cancer OR Cancer, Mammary OR Cancers, Mammary OR Mammary Cancers OR Malignant Neoplasm of Breast OR Breast Malignant Neoplasm OR Breast Malignant Neoplasms OR Malignant Tumor of Breast OR Breast Malignant Tumor OR Breast Malignant Tumors OR Cancer of Breast OR Cancer of the Breast OR Mammary Carcinoma, Human OR Carcinoma, Human Mammary OR Carcinomas, Human Mammary OR Human Mammary Carcinomas OR Mammary Carcinomas, Human OR Human Mammary Carcinoma OR Mammary Neoplasms, Human OR Human Mammary Neoplasm OR Human Mammary Neoplasms OR Neoplasm, Human Mammary OR Neoplasms, Human Mammary OR Mammary Neoplasm, Human OR Breast Carcinoma OR Breast Carcinomas OR Carcinoma, Breast OR Carcinomas, Breast) |
| #2 | TS=(Prognosis OR Prognosis OR Prognoses OR Prognoses OR Prognostic Factors OR Prognostic Factors OR Prognostic Factor OR Prognostic Factor OR Factor, Prognostic OR Factor, Prognostic OR Factors, Prognostic OR Factors, Prognostic OR Overall survival OR Overall survival OR OS OR OS OR metastasis OR metastasis OR Disease-Free Survival OR Disease-Free Survival OR Disease Free Survival OR Disease Free Survival OR Survival, Disease-Free OR Survival, Disease-Free OR Survival, Disease Free OR Survival, Disease Free OR DFS OR DFS OR Progression-Free Survival OR Progression-Free Survival OR Progression Free Survival OR Progression Free Survival OR Survival, Progression-Free OR Survival, Progression-Free OR Event-Free Survival OR Event-Free Survival OR Event Free Survival OR Event Free Survival) |
| #1 | TS=(Radiomics OR radiomic OR radiogenomic OR radiomics-based OR radiomic signature) |
