# Supplementary material for: Predictive value of radiomics-based machine learning for the disease-free survival in breast cancer: a systematic review and meta-analysis
Source: Front Oncol. 2023 Aug 16;13:1173090. doi: 10.3389/fonc.2023.1173090 (PMC10469000; doi:10.3389/fonc.2023.1173090)
Supplement: Supplementary file 3 [file Table_2.docx]

**Table S2**

Quality assessment of included studies via RQS.

| Author | Year | Q1 | Q2 | Q3 | Q4 | Q5 | Q6 | Q7 | Q8 | Q9 | Q10 | Q11 | Q12 | Q13 | Q14 | Q15 | Q16 | total |
| --- | --- | --- | --- | --- | --- | --- | --- | --- | --- | --- | --- | --- | --- | --- | --- | --- | --- | --- |
| Yunfang Yu | 2020 | +1 | +1 | -1 | -1 | +3 | +1 | +1 | +1 | +1 | +1 | 0 | +2 | +2 | +2 | -1 | +3 | 16 |
| Lang Xiong | 2021 | +1 | +1 | -1 | +1 | +3 | +1 | +1 | +1 | +1 | +1 | 0 | +2 | +2 | +2 | -1 | +3 | 18 |
| Ling Zhang | 2020 | +2 | +1 | +1 | +1 | +3 | +1 | +1 | +1 | +2 | +2 | 0 | +2 | +2 | +2 | +1 | +4 | 26 |
| Bingqing Xia | 2021 | +2 | +1 | +1 | +1 | +3 | +1 | +1 | +1 | +1 | +1 | 0 | +3 | +2 | -2 | -1 | +3 | 18 |
| Feihong Yu | 2021 | +1 | +1 | -1 | +1 | +3 | +1 | +1 | +1 | +2 | +2 | 0 | +3 | +2 | +2 | +1 | +3 | 23 |
| Sungwon Kim | 2020 | +6 | +1 | +1 | -1 | +3 | +1 | -1 | +1 | +1 | +1 | 0 | +2 | +2 | -2 | -1 | +3 | 17 |
| Hwan-ho Cho | 2022 | +2 | +1 | -1 | +1 | +3 | +1 | +1 | +1 | +1 | +1 | 0 | +3 | +2 | -2 | -1 | +4 | 17 |
| Qin Li | 2020 | +2 | +1 | +1 | -1 | +3 | +1 | +1 | +1 | +2 | +2 | 0 | +2 | -2 | +2 | +1 | +3 | 19 |
| Xian Jiang | 2020 | +1 | +1 | +1 | +1 | +3 | +1 | -1 | +1 | +1 | +1 | 0 | +2 | +2 | +2 | -1 | +3 | 18 |
| Haoyu Wang | 2022 | +1 | +1 | -1 | -1 | +3 | +1 | +1 | +1 | +2 | +2 | 0 | +3 | -2 | +2 | -1 | +3 | 15 |
| Hyunjin Park | 2018 | +2 | +1 | +1 | -1 | +3 | +1 | +1 | +1 | +2 | +1 | 0 | +2 | +2 | +2 | -1 | +3 | 20 |
| Xuanyi Wang | 2022 | +1 | +1 | -1 | -1 | +3 | +1 | +1 | +1 | +1 | +1 | 0 | +3 | +2 | +2 | -1 | +3 | 17 |
| Jeongmin Lee | 2022 | +1 | +1 | -1 | +1 | +3 | +1 | -1 | +1 | +1 | +1 | 0 | +2 | +2 | +2 | -1 | +3 | 16 |

Q1: Image protocol quality; Q2: Multiple segmentation; Q3: Phantom study; Q4: Imaging at multiple time points; Q5: Feature reduction or adjustment for multiple testing; Q6: Multivariable analysis; Q7: Biological correlates; Q8: cut-off analyses; Q9: Discriminationg statistics; Q10: Calibration statistics; Q11: Prospective study; Q12: Validation; Q13: Comparision to “gold standard”; Q14: Potential clinical application; Q15: Cost-effectiveness analysis; Q16: Open science and data.
